# Supplementary material for: Pain Assessment in Cattle by Use of Numerical Rating and Visual Analogue Scales—A Systematic Review and Meta-Analysis
Source: Animals (Basel). 2024 Jan 22;14(2):351. doi: 10.3390/ani14020351 (PMC10812761; doi:10.3390/ani14020351)
Supplement: Supplementary file 1 [file animals-14-00351-s001.zip › Supplemental S1_PRISMA P checklist.pdf]

Table S1: PRISMA-P checklist for the systematic review "Substance P concentrations in adult cattle and calves during different painful procedures and conditions – a systematic review" according to Shamseer et al. (2015) [25].

Section and Topic	Item Nr.	Checklist Item
**Administrative Information**		
Title		
Identification	1a	Identify the report as a protocol of a systematic review<br><br>- Protocol for a systematic review to evaluate pain assessment in veterinarians, producers, farmers, and other people handling cattle presented with specific conditions and procedures, using either a Numerical Rating or Visual Analogue Scale
Update	1b	If the protocol is for an update of a previous systematic review, identify as such<br><br>- This is the first attempt for a systematic review covering the above-mentioned topic, and no update of a previously conducted systematic review.
Registration	2	If registered, provide the name of the registry (such as PROSPERO) and registration number<br><br>- Not applicable; according to its website, PROSPERO can only be used to studies in human medicine/human patients.
Authors		
Contact	3a	Provide name, institutional affiliation, e-mail address of all protocol authors; provide physical mailing address of corresponding author<br><br>- Corresponding author: Theresa Tschoner<br><br>t.tschoner@lmu.de<br><br>Melanie Feist: M.Feist@lmu.de<br><br>Yury Zablotksi: Y.Zablotski@med.vetmed.uni-muenchen.de

		Author affiliation:<br><br>Clinic for Ruminants with Ambulatory and Herd Health Services, Centre for Clinical Veterinary Medicine, LMU Munich, Sonnenstrasse 16, 85764 Oberschleissheim, Germany<br><br><br>Kristina Müller: K.Mueller@massey.ac.at<br><br>Author affiliation:<br><br>Massey University, School of Veterinary Science, Private Bag 11 222, Palmerston North, 4474 New Zealand;
Contributions	3b	Describe contributions of protocol authors and identify the guarantor of the review<br><br>- TT is the guarantor; TT drafted the manuscript, TT, KM, and MF developed the search strategy and the criteria for the selection. TT selected the titles, TT, KM, and MF screened the abstracts and selected the references for full-text review, TT did the full text review. All authors read, proved feedback, and approved the final manuscript.
Amendments	4	If the protocol represents an amendment of a previously completed or published protocol, identify as such and list changes; otherwise, state plan for documenting important protocol amendments<br><br>- This protocol doesn't represent an amendment of a previously completed/published protocol. If amendments of this protocol are necessary, they will be included with the date of the amendment, and a description thereof.
Support		
Sources	5a	Indicate sources of financial or other support for the review<br><br>- There was no financial support of this study itself. TT is founded with a grant by the Deutsche Forschungsgesellschaft (DFG, grant number 505835300). Salary was ensured to MF, KM, and YZ.

Sponsor	5b	Provide name for the review funder and/or sponsor<br><br>- Not applicable.
Role of sponsor or funder	5c	Describe roles of funder(s), sponsor(s), and/or institution(s), if any, in developing the protocol<br><br>- There was no role of funder(s) in the development of the protocol.
**Introduction**		
Rationale	6	Describe the rationale for the review in the context of what is already known<br><br>Pain evaluation and assessment in cattle is difficult, and largely depends on the people handling the animals, like veterinarians, farmers, and producers. Pain assessment by individuals is subjective, and studies showed that age, gender, and empathy influence the way pain is assessed in cattle. Surveys about pain assessment in cattle have been published, with respondents presented with different painful procedures and conditions, which they needed to rank using either a Numerical Rating or Visual Analogue Scale. Even if median ranks are similar across these studies, the ranges vary from lowest to highest. To this day, no systematic review or meta-analysis about the assessment of painful conditions and procedures using rating scales has been been published. Therefore, the objectives of this systematic review were to 1) describe and compare median pain scores and their ranges awarded by veterinarians and producers using either a Numerical Rating or a Visual Analogue Scale, 2) compare these scores with a meta-analysis, and 3) assess pain management in respondents if stated. The aim of this review is to contribute to the current knowledge about pain assessment in cattle, and the possible differences between veterinarians and producers.
Objectives	7	Provide an explicit statement of the question(s) the review will address with reference to participants, interventions, comparators, and outcomes (PICO)<br><br>- The aim of this systematic review if to compare pain scores as awarded by people handling cattle over different countries and years for different painful conditions and procedures in cattle (cows and calves).
**Methods**		

Eligibility criteria	8	Specify the study characteristics (such as PICO, study design, setting, time frame) and report characteristics (such as years considered, language, publication status) to be used as criteria for eligibility for the review<br><br>- All studies published in English or German about providing pain scores using either a Numerical Rating or Visual Analogue Scale for different painful procedures and conditions in cattle will be selected. The authors expect mainly experimental studies, but will not exclude any study type, to include as many articles as possible. The authors will define no time frame, the literature search will be performed to the date of the 11th of September 2023. Studies will be included if the following 8 questions can be answered with "yes":<br><br>1) Can the full text be obtained?<br>2) Is the full text written in English or German<br>3) Is the study population either veterinarians, producers, or farmers?<br>4) Is the study design a survey or a questionnaire?<br>5) Is the questionnaire or survey about the assessment of painful conditions/procedures?<br>6) Is either a Numerical Rating or Visual Analogue Scale used for pain assessment?<br>7) Is the questionnaire about cattle?<br>8) Is the article peer-reviewed?
Information sources	9	Describe all intended information sources (such as electronic databases, contact with study authors, trial registers or other grey literature sources) with planned dates of coverage<br><br>- The literature search will be conducted with the following data bases: PubMed, Medline, Agricola.
Search strategy	10	Present draft of search strategy to be used for at least one electronic database, including planned limits, such that it could be repeated<br><br>To identify studies with a study population of veterinarians, farmers, or othere people working with cattle: (veterinar* OR farm* OR produc* OR livestock* OR clinic* OR practition* OR caretak*) AND:

		2. To identify studies done on cattle: (cattle OR cow OR calves OR calf OR dairy OR beef OR bovine) AND:<br><br>3. To identify all studies where a questionnaire was done: (survey OR question* OR attitud* OR opinion*) AND:<br><br>4. To identify all studies with surveys done on pain assessment or management: (pain* OR analges*).
<u>Study records</u>		
Data management	11a	Describe the mechanism(s) that will be used to manage records and data throughout the review<br><br>- We will use Microsoft Excel and Endnote to manage the records and data.
Selection process	11b	State the process that will be used for selecting studies (such as two independent reviewers) through each phase of the review (that is, screening, eligibility and inclusion in meta-analysis)<br><br>- After deduplication of titles (TT), TT will screen all titles. Afterwards, TT, MF, and KM will independently and blindly screen abstracts of the articles included after the search and check for the inclusion criteria. Afterwards, all full texts of the included articles will be obtained and screened by TT and will be included in the systematic review if they meet the above mentioned inclusion criteria. Neither author will be blinded to the title, authors, or journal in which the reference has been published. If full texts can't be retrieved, the publishing authors will be contacted.
Data collection process	11c	Describe planned method of extracting data from reports (such as piloting forms, done independently, in duplicate), any processes for obtaining and confirming data from investigators<br><br>- Data extraction will be done by TT after assessment of full texts. Data regarding primary author, year of publication, country, group and number of participants, return rate, demographic data of participants, pain scale used, painful condition and procedures assessed in either adult cows of calves, assessment of necessity and/or use of analgesics, and funding information

		will be extracted. Data will be extracted and collected using Miscrosoft Excel.
Data items	12	List and define all variables for which data will be sought (such as PICO items, funding sources), any pre-planned data assumptions and simplifications<br><br>- Data extraction will include information on primary author, year of publication, country, group and number of participants, return rate, demographic data of participants, pain scale used, painful condition and procedures assessed in either adult cows of calves, assessment of necessity and/or use of analgesics, and funding information .Data will be extracted and collected using Miscrosoft Excel. Data and information will be checked for the suitability to perform a meta-analysis.
Outcomes and prioritization	13	List and define all outcomes for which data will be sought, including prioritization of main and additional outcomes, with rationale<br><br>- The primary outcome will be pain assessment for different procedures and conditions in cattle, done using either a Numerical Rating or Visual Analogue Scale. Data will be sought for the outcomes pain scores awarded by using either a Numerical Rating or Visual Analogue Scale, painful conditions and procedures, people awarding the pain scores (veterinarians, producers, farmers, or other), and animal population (calf or adult cow).
Risk of bias in individual studies	14	Describe anticipated methods for assessing risk of bias of individual studies, including whether this will be done at the outcome or study level, or both; state how this information will be used in data synthesis<br><br>- If a meta-analysis can be done, potential bias across studies will be presented graphically. If not, it will be presented in the sections “results” and “discussion”.
Data synthesis	15a	Describe criteria under which study data will be quantitatively synthesised<br><br>- If the studies included in this systematic review are homogenous in study design and comparator, and if they provide statistical information about pain scores which can be retrieved from the data, a meta-analysis will be performed.

	15b	If data are appropriate for quantitative synthesis, describe planned summary measures, methods of handling data and methods of combining data from studies, including any planned exploration of consistency (such as I^2, Kendall's τ)<br><br>- If data are appropriate for a quantitative synthesis, Odds ratios (OR) and their 95% confidence intervals will be calculated.
	15c	Describe any proposed additional analyses (such as sensitivity or subgroup analyses, meta-regression)<br><br>- Not planned.
	15d	If quantitative synthesis is not appropriate, describe the type of summary planned<br><br>- If a quantitative synthesis can't be performed, a verbal summary of the findings of the systematic review will be done, including the assessment of pain by using pain scales, and comparison of these in a table for different populations of people handling cattle, age of animals, and procedures/conditions.
Meta-bias(es)	16	Specify any planned assessment of meta-bias(es) (such as publication bias across studies, selective reporting within studies)<br><br>- If a meta-analysis can be done, potential bias across studies will be presented graphically. If not, it will be presented in the sections "results" and "discussion".
Confidence in cumulative evidence	17	Describe how the strength of the body of evidence will be assessed (such as GRADE)<br><br>- Studies will be included in the systematic review if the following 4 questions can be answered with "yes", as described by [29]:<br><br>1) Can the full text be obtained?<br>2) Is the full text written in English or German<br>3) Is the study population either veterinarians, producers, or farmers?<br>4) Is the study design a survey or a questionnaire?

		5) Is the questionnaire or survey about the assessment of painful conditions/procedures?<br>6) Is either a Numerical Rating or Visual Analogue Scale used for pain assessment?<br>7) Is the questionnaire about cattle?<br>8) Is the article peer-reviewed?<br><br>Apart from that, no other reporting guidelines will be used.
