# Supplementary material for: Pain Assessment in Cattle by Use of Numerical Rating and Visual Analogue Scales—A Systematic Review and Meta-Analysis
Source: Animals (Basel). 2024 Jan 22;14(2):351. doi: 10.3390/ani14020351 (PMC10812761; doi:10.3390/ani14020351)
Supplement: Supplementary file 1 [file animals-14-00351-s001.zip › Supplemental S2_Material and Methods.pdf]

Table S2: Findings of Material and Methods section for 16 references about the assessment of pain in cattle, including funding information. Reference (**Ref**) of the according studies is indicated.

Year	Author	Ref.	Animal Group	Survey Form	Funding Information
2006	Huxley and Whay	[9]	- Adult Cattle<br>- Calves	- Paper	- Boehringer Ingelheim
2007	Hewson et al.[1]	[10]	- Adult Cattle<br>- Calves	- Paper	- Animal Welfare Foundation of Canada<br>- Sir Dunn Animal Welfare Center, Atlantic Veterinary College, University of Prince Edward Island
2009	Kielland et al.	[15]	- Adult Cattle<br>- Calves	- Online	- None stated
2009	Laven et al.	[12]	- Adult Cattle<br>- Calves	- Paper	- Boehringer Ingelheim
2010	Kielland et al.	[19]	- Adult Cattle<br>- Calves	- Paper<br>- Online	- Research Council of Norway (15%)<br>- Agricultural Agreement Research Fund (16%)<br>- Foundation for Research Levy on Agricultural Products (69%)
2012	Thomsen et al.	[13]	- Adult Cattle	- Paper	- Not stated explicitly<br>- Boehringer Ingelheim and Danish Cattle Federation mentioned in Achnowledgments
2013	Becker et al.	[30]	- Adult Cattle	- Online	- Federal Veterinary Office BVET
2013	Lorena et al.[2]	[31]	- Not stated	- Paper<br>- Online	- None stated
2013	Wikman et al.	[20]	- Adult Cattle<br>- Calves	- Paper	- Ministry of Agricultural and Forestry in Finland<br>- Finnish Veterinary Foundation<br>- Swedish Cultural Fund<br>- Swedish Ostrobothnian Society<br>- Sponsors: Valio Ltd., Vetman Ltd., Vking Genetics, Osuuskunta Maitosuomi
2014	Norring et al.	[22]	- Adult cattle<br>- Calves	- Paper<br>- Online	- Ministry of Agricultural and Forestry in Finland
2015	Hokkanen et al.	[32]	- Calves	- Paper	- Ministry of Agricultural and Forestry in Finland<br>- Valio Ltd., Vetman Ltd., Vking Genetics, Osuuskunta Maitosuomi<br>- Hämäläis-Osakunta personal grant to Tanja Korhonen

Continuing Table S2: Findings of Material and Methods section for 16 references about the assessment of pain in cattle, including funding information. Reference (**Ref**) of the according studies is indicated.

Year	Author	Ref.	Animal Group	Survey Form	Funding Information
2017	Remnant et al.	[1]	- Adult Cattle<br>- Calves	- Paper<br>- Online	- Boehringer Ingelheim
2020	Tschoner et al.	[11]	- Adult Cattle<br>- Calves	- Paper<br>- Online	- Bavarian Ministry of Food, Agriculture and Forestry<br>- Boehringer Ingelheim<br>- Pfizer<br>- Merial
2021	Tschoner et al.	[14]	- Adult Cattle<br>- Calves	- Paper	- Bavarian Ministry of Food, Agriculture and Forestry<br>- Boehringer Ingelheim<br>- Pfizer<br>- Merial
2022	Van Dyke et al.	[33]	- Calves	- Online	- No external Funding
2022	Shi et al.	[16]	- Adult cattle<br>- Calves	- Online	- Boehringer Ingelheim Animal Health (Shanghai, China)

[1] Survey conducted on pain management in cattle, horses, and pigs

[2] Survey conducted on pain management in cattle and horses
