# Supplementary material for: Pain Assessment in Cattle by Use of Numerical Rating and Visual Analogue Scales—A Systematic Review and Meta-Analysis
Source: Animals (Basel). 2024 Jan 22;14(2):351. doi: 10.3390/ani14020351 (PMC10812761; doi:10.3390/ani14020351)
Supplement: Supplementary file 1 [file animals-14-00351-s001.zip › Supplemental S3 Procedures in Cattle.pdf]

Supplemental Table S3: Pain scoring for different procedures presented to participants of surveys about pain assessment in adult cattle, under the assumption that no analgesics are used. Use of analgesics was not stated for [13,18,19,21]. Pain scoring was either done by veterinarians (V), veterinary students (VS), farmers (F), producers (and frontline staff, P), and/or claw trimmers (C) by use of a Numerical Rating (NRS, ranging from either 0 – 10 or 1 – 10) or Visual Analogue Scale (VAS, ranging from 0 – 10), or both. [19] described using a NRS in the Material and Methods section, but indicated use of a VAS in the results section and is therefore included as VAS. Ranges are included in brackets if indicated in the references. If procedures were not presented in the respective reference, this is indicated as -.

	**NRS (0 – 10)**						**NRS (1 – 10)**								**VAS (0 – 10)**		
	[9]	[12]	[22][1]	[11]	[14]	[16][1]	[10][1]	[15]	[13][2]		[30]			[1]	[15]	[19]	[20]
Group	V	V	V/VS	V	F	V/F/P	V	VS	V	F	V	F	C	V	VS	F	P
Artificial Insemination	-	-	-	1<br>(0 – 7)	0<br>(0 – 8)	-	-	-	-		-			-	-	-	-
Caesarean section	9<br>(1 – 10)	9<br>(4 – 10)	-	9<br>(0 – 10)	9<br>(0 – 10)	8.6 ± 2.12	8	-	-		-			9<br>(5 – 10)	-	-	-
Claw amputation	10<br>(2 – 10)	10<br>(5 – 10)	-	9<br>(5 – 10)	9<br>(5 – 10)	-	-	-	-		10<br>(8 – 10)	10<br>(7 – 10)	10<br>(8 – 10)	10<br>(8 – 10)	-	-	-
Debriding of a digital dermatitis lesion	6<br>(1 – 10)	-	-	-	-	-	-	-	-		-			7<br>(2 – 10)	-	-	-
De-horning[3]	8<br>(2 – 10)	8<br>(3 – 10)	-	8<br>(0 – 10)	8<br>(0 – 10)	7.1 ± 2.52	7.4	4<br>(1 – 9)	-		-			8<br>(2 – 10)	3<br>(1 – 8)	5.1<br>(0 – 10)	-
Extirpation of eye bulb	-	-	-	9<br>(3 – 10)	10<br>(3 – 10)	-	-	-	-		-			-	-	-	-
Fetotomy	-	-	-	7<br>(1 – 10)	7/8<br>(1 – 10)	-	-	-	-		-			-	-	-	-
Laparoscopic fixation of LDA	-	-	-	-	8<br>(2 – 10)	-	-	-	-		-			-	-	-	-
Laparotomy	-	-	-	8<br>(2 – 10)	9<br>(2 – 10)	-	-	-	-		-			-	-	-	-
Left displacement of the abomasum surgery	9<br>(2 – 10)	9<br>(3 – 10)	-	-	-		-	-	-		-			8<br>(3 – 10)	-	-	-
Needle prick[4]	-		2.5 ± 1.87	-	-		-	-	-		-				-	-	-
Omentopexy	-	-	-	-	9<br>(0 – 10)		-	-	-		-				-	-	-
Rectal examination	-	-	-	1/2	1		-	-	-		-				-	-	-

				(0 – 7)	(0 – 9)											
Removal of retained fetal membranes	-	-	-	3 (0 – 8)	5 (0 – 8)		-	4 (1 – 9)	-	-				2 (0 – 4)	2.4 (0 – 7)	-
Treatment of interdigital hyperplasia[5]	-	-	-	8 (2 – 10)	7 (2 – 10)		-	-	-	8 (4 – 10)	8 (3 – 10)	9 (3 - 10)		-	-	-
Treatment of a sole ulcer[5]	6 (1 – 10)	-	-	7 (1 – 10)	7 (1 – 10)		-	-	-	8 (3 - 10)	7 (3 - 10)	7 (1 – 10)	7 (2 – 10)	-	-	-
Treatment of white line abscess[5]	-	4 (1 – 10)	-	-	-		-	-	-	8 (2 – 10)	7 (3 - 10)	7 (1 – 10)		-	-	-

[1] Mean values including standard deviation where indicated; [2] median as well as mean values presented in article, median values were included in the table; [3] horns > 8 cm long for Huxley and Whay (2006), Laven et al. (2009); in cattle over 6 months of age for Hewson et al. (2007); [4] fully grown cattle, into the shoulder muscle for Norring et al. (2014); [5] excision for Becker et al. (2013);
