# Supplementary material for: Pain Assessment in Cattle by Use of Numerical Rating and Visual Analogue Scales—A Systematic Review and Meta-Analysis
Source: Animals (Basel). 2024 Jan 22;14(2):351. doi: 10.3390/ani14020351 (PMC10812761; doi:10.3390/ani14020351)
Supplement: Supplementary file 1 [file animals-14-00351-s001.zip › Supplemental S4 Conditions in Cattle.pdf]

Supplemental Table S4: Pain scoring for different conditions presented to participants of surveys about pain assessment in adult cattle, under the assumption that no analgesics are used. Use of analgesics was not stated for [13,18,19,21]. Pain scoring was either done by veterinarians (V), veterinary students (VS), farmers (F), and/or producers (and frontline staff, P) by use of a Numerical Rating (NRS, ranging from either 0 – 10 or 1 – 10) or Visual Analogue Scale (VAS, ranging from 0 – 10), or both. [19]described using a NRS in the Material and Methods section, but indicated use of a VAS in the results section and is therefore included as VAS. Ranges are included in brackets if indicated in the references. If procedures were not presented in the respective reference, this is indicated as -.

	**NRS (0 – 10)**						**NRS (1 – 10)**								**VAS (0 – 10)**		
	[9]	[12]	[22][1]	[11]	[14]	[16][1]	[10][1]	[15]	[13][2]		[30]			[1]	[15]	[19]	[20]
Group	V	V	V/VS	V	F	V/F/P	V	VS	V	F	V	F	C	V	VS	F	P
Abomasal displacement	-	-	7.3 ± 1.9	-	-	7.4 ± 2.18	-	-	-		-			-	-	-	8
Acute metritis[3]	4 (1 – 10)	-	-	5 (0 – 10)	5 (0 – 10)	-	-	7 (2 – 10)	6	6	-			5 (1 – 10)	4 (1 – 5)	-	-
Acute mastitis[4]	-	-	7.3 ± 1.4	-	-	-	-				-			-	-	7.6 (0 – 10)	8
Acute toxic (E. Coli) mastitis[5]	7 (1 – 10)	8 (4 – 10)	-	7 (1 – 10)	7 (1 – 10)	7 ± 2.2	-	7 (4 – 10)	9	9	-			7 (3 – 10)	7 (3 - 10)	-	-
After removal of retained fetal membranes	-	-	-	-	-	-	-	4 (1 – 10)	-		-			-	4 (1 - 10)	2.4 (0 – 7)	-
Calving	-	-	-	-	-	8.5 ± 1.99	-		-		-			-	-	-	-
Chronic mastitis (clots in milk only)	-	-	-	1 (0 – 8)	1 (0 – 8)	-	-	-	-		-			-	-	-	-
Corneal ulcer	-	-	-	-	-	-	5.5	-	-		-			-	-	-	-
Decubitus	-	-	-	4/5 (0 – 10)	4/5 (0 – 10)	-	-	-	-		-			-	-	-	-
Digital Dermatitis	6 (2 – 10)	-	-	7 (2 – 10)	7 (2 – 10)	-	-	-	7	7	-			6 (2 – 9)	-	-	-
Dystocia[6]	7 (2 – 10)	7 (2 – 10)	7.3 ± 1.7	8 (2 – 10)	8 (2 – 10)	9.0 ± 1.83	5.3	8 (1 – 10)	-		-			7 (3 – 10)	8 (2 – 10)	-	-
Endometritis	-	-	-	-	-	5.9 ± 2.62	-	-	-		-			-	-	-	-
Footrot	-	5 (2 – 9)	-	-	-	-	-	-	-		-			-	-	-	-
Fracture of long bone[7]	-	-	-	8 (3 – 10)	8 (3 – 10)	8.4 ± 2.08	-	-	-		-			-	-	-	-

Fracture of the horn	-	-	-	6 (0 – 10)	6 (0 – 10)	-	-	-	-		-	-	-	-	-
Fracture of tuber coxae[8]	7 (2 – 10)	8 (2 – 10)	-	-	-	-	-	9 (3 – 10)	8	8	-	8 (4 – 10)	8 (4 – 10)	-	-
Hock with hair loss	3 (1 – 10)	-	-	-	-	-	-	-	-		-	3 (1 – 9)	-	-	-
Hoof disasease	-	-	-	-	-	6.9 ± 2.18	-	-	-		-	-	-	-	-
Hypocalcemia[9]	-	-	-	1 (0 – 10)	1 (0 – 10)	-	-	5 (1 – 10)	-		-	-	5 (1 – 10)	3.3 (0 – 9)	-
Infectious disease	-	-	-	-	-	4.5 ± 2.91	-	-	-		-	-	-	-	-
Injuries on hock[10]	-	-	-	-	-	-	-	4 (1 - 8)	-		-	-	4 (1 – 7)	2.9 (0 – 9)	-
Interdigital necrobacillosis	-	-	-	-	-	-	-		8	8	-	-	-	-	-
Intertrigo	-	-	-	4 (0 – 10)	4 (0 – 10)	-	-	-	-		-	-	-	-	-
Ketosis	-	-	-	1 (0 – 10)	1 (0 – 10)	-	-	4 (1 – 10)	-		-	-	4 (1 - 10)	-	-
Laminitis	-	-	-	8 (2 – 10)	8 (2 – 10)	-	-	7 (3 – 10)	-		-	-	7 (1 -- 10)	5.7 (1 – 10)	-
Left displaced abomasum	3 (1 – 10)	6 (1 – 10)	-	5 (0 – 10)	5 (0 – 10)	-	-	6 (2 - 10)	5	6	-	4 (1 – 10)	7 (1 – 10)	-	-
Loss of nose ring	-	-	-	6 (1 – 10)	6 (1 – 10)	-	-	-	-		-	-	-	-	-
Mastitis (clots in milk only)[11]	3 (1 – 10)	3 (1 – 9)	-	-	-	3.4 ± 2.65	-	4 (1 - 10)	2	3	-	4 (1 – 8)	5 (1 - 10)	-	-
Moderate mastitis	-	-	-	-	-	5.1 ± 2.24	-	-	-		-	-	-	-	-
Neck calluses	2 (1 – 7)	-	-	3 (0 – 9)	3 (0 – 9)	-	-	4 (1 – 9)	3	4	-	3 (1 – 7)	4 (1 – 9)	-	-
Nutritional deficiency disease	-	-	-	-	-	3.6 ± 2.8	-	-	-		-	-	-	-	-
Oesophageal obstruction	-	-	-	-	-	5.9 ± 2.36	-	-	-		-	-	-	-	-
Open teat injury	-	-	-	6 (0 – 10)	6 (0 – 10)	-	-	-	-		-	-	-	-	-
Parasitic disease	-	-	-	-	-	4.1 ± 2.57	-	-	-		-	-	-	-	-
Postpartum paralysis	-	-	-	-	-	5.9 ± 3.02	-	-	-		-	-	-	-	-

Right displaced abomasum	-	-	-	6 (0 – 10)	6 (0 – 10)	-	-	-	-		-	-	-	-	-
Ruminal acidosis	-	-	-	-	-	5.3 ± 2.62	-	-	-		-	-	-	-	-
Rupture of muscle	-	-	-	8 (2 – 10)	8 (2 – 10)	-	-	-	-		-	-	-	-	-
Septic Arthritis/Polyarthritis	-	-	-	8 (3 – 10)	8 (3 – 10)	-	-	-	-		-	-	-	-	-
Severe tympany in cattle[12]	-	-	7.9 ± 1.6	-	-	6.1 ± 2.18	-	-	-		-	-	-	-	9
Sole ulcer	-	-	-	8 (2 – 10)	8 (2 – 10)	-	-	7 (4 – 10)	-		-	-	6 (1 – 9)	7.1 (0 – 10)	-
Swollen hock	5 (1 – 10)	6 (2 – 10)	-	-	-	-	-	5 (1 – 9)	5	5	-	5 (2 – 9)	5 (1 – 9)	-	-
Teat tramping in cows[13]	-	-	7.4 ± 1.7	-	-	-	-	-	-		-	-	-	-	-
Tissue injuries following birth	-	-	-	5 (0 – 10)	5 (0 – 10)	-	-	-	-		-	-	-	-	-
Uterine torsion	-	-	-	6 (0 – 10)	6 (0 – 10)	-	-	-	-		-	-	-	-	-
Traumatic pericarditis	-	-	-	-	-	7.8 ± 2.25	-	-	-		-	-	-	-	-
Uterine prolapse[14]	-	-	6.9 ± 2.0	5 (0 – 10)	5 (0 – 10)	7.9 ± 2.3	-	-	-		-	-	-	-	8
Uveitis[15]	6 (1 – 10)	-	-	5 (0 – 10)	5 (0 – 10)	-	-	6 (2 – 10)	8	4	-	6 (2 – 10)	5 (1 – 10)	-	-
Vaginal prolapse	-	-				6.3 ± 2.66	-	-	-		-	-	-	-	-
White line disease[16]	7 (1 – 10)	-	-	-	-	-	-	-	-		-	7 (2 – 10)	-	-	-

[1] Mean values including standard deviation where indicated; [2] median as well as mean values presented in reference, median values were included in the table; [3] puerperal metritis for Tschoner et al. (2020, 2021), metritis for Remnant et al. 2017; [4] fever 41°C, lumps in milk, hard udder for Norring et al. (2014); [5] *Escherichia coli* mastitis for Huxley and Whay (2006), serious mastitis for Kielland et al. (2009; 2010); severe mastitis for Shi et al. (2022); [6] fetal-maternal disproportion requiring traction alone for Huxley and Whay (2006), Laven et al. (2009), Tschoner et al. (2020, 2021); strong pull assistance for Norring et al. (2014); [7] fracture for Shi et al. (2022); [8] one sided for Kielland et al., (2010); [9] milk fever for Kielland et al. (2009; 2010); [10] skin lesions on hock for Kielland et al. (2010); [11] mastitis for Thomsen et al., (2012), mild mastitis for Shi et al. (2022) [12] ruminal bloat for Shi et al. (2022); [13] teat broken at the roof for Wikman et al. (2013); [14] uterine eversion for Tschoner et al. (2020, 2021); [15] Eye infection for Kielland et al. (2009; 2010); [16] with subsole abscess for Huxley and Whay (2006);
