# Supplementary material for: Pain Assessment in Cattle by Use of Numerical Rating and Visual Analogue Scales—A Systematic Review and Meta-Analysis
Source: Animals (Basel). 2024 Jan 22;14(2):351. doi: 10.3390/ani14020351 (PMC10812761; doi:10.3390/ani14020351)
Supplement: Supplementary file 1 [file animals-14-00351-s001.zip › Supplemental S5 Procedures in Calves.pdf]

Supplemental Table S5: Pain scoring for different procedures presented to participants of surveys about pain assessment in calves, under the assumption that no analgesics are used. Use of analgesics was not stated for [13,18,19,21]. Pain scoring was either done by veterinarians (V), veterinary students (VS), farmers (F), and/or producers (and frontline staff, P) by use of a Numerical Rating (NRS, ranging from either 0 – 10 or 1 – 10) or Visual Analogue Scale (VAS, ranging from 0 – 10), or both. [19] described using a NRS in the Material and Methods section, but indicated use of a VAS in the results section and is therefore included as VAS. Ranges are included in brackets if indicated in the references. If procedures were not presented in the respective reference, this is indicated as -.

	**NRS 0 - 10**						**NRS (1 – 10)**			**VAS (0 – 10)**		
	[9]	[12]	[22][1]	[11]	[14]	[16][1]	[10][1]	[15]	[1]	[15]	[19]	[20]
Group	V	V	V/VS	V	F	V/F/P	V	VS	V	VS	F	P
Castration[2] up to 6 months	-	-	-	-	-	-	4.9	-	-	-	-	-
Castration[2] over 6 months	-	-	-	-	-	-	5.9	-	-	-	-	-
Castration (Burdizzo)	7<br>(2 – 10)	6<br>(2 – 10)	-	9<br>(2 – 10)	9<br>(2 – 10)	-	-	-	6<br>(2 – 10)	-	-	-
Castration (Rubber Ring)	6<br>(1 – 1)	5<br>(1 – 10)	-	-	-	-	-	-	6<br>(2 – 10)	-	-	-
Castration (Surgical)	6<br>(2 – 10)	8<br>(2 – 10)	-	9<br>(1 – 10)	9<br>(1 – 10)	7.8 ± 2.32	-	-	7<br>(2 – 10)	-	-	-
Dehorning[3]	-	-	-	8<br>(1 – 10)	8<br>(1 – 10)	-	6.8	-	-	-	-	-
Dehorning over 6 months	-	-	-	-	-	-	7.4	-	-	-	-	-
Disbudding	7<br>(2 – 10)	8<br>(3 – 10)	9 ± 1.2	-	-	7.6 ± 2.32	-	-	7<br>(2 – 10)	-	-	9
Disbudding (caustic paste)	-	-	-	-	-	5.6 ± 2.52	-	-	-	-	-	-
Disbudding with analgesics[4]	-	-	2.4 ± 1.8	-	-	-	-	-	-	-	-	-
Ear tagging	-	-	-	4<br>(0 – 10)	4<br>(0 – 10)	-	-	-	-	-	-	-
Laparotomy	-	-	-	9<br>(1 – 10)	9<br>(1 – 10)	-	-	-	-	-	-	-
Repair of distal limb fracture	-	10<br>(2 – 10)	-	-	-	-	-	-	-	-	-	-
Tenotomy of contracted tendons	-	-	-	8<br>(2 – 10)	8<br>(2 – 10)	-	-	-	-	-	-	-
Umbilical hernia surgery[5]	8<br>(2 – 10)	8<br>(3 – 10)	-	9<br>(1 – 10)	9<br>(1 – 10)	6.8 ± 2.32	7.3	-	8<br>(4 – 10)	-	-	-

[1] mean values including standard deviation where indicated; [2] Hewson et al. (2007) did not distinguish between methods of castration; [3] in calves up to 6 months for Hewson et al. (2007); [4] pain during burning for Wikman et al. (2013) and Norring et al. (2014); [5] in calves up to 3 months for Hewson et al. (2007);
