# Supplementary material for: Pain Assessment in Cattle by Use of Numerical Rating and Visual Analogue Scales—A Systematic Review and Meta-Analysis
Source: Animals (Basel). 2024 Jan 22;14(2):351. doi: 10.3390/ani14020351 (PMC10812761; doi:10.3390/ani14020351)
Supplement: Supplementary file 1 [file animals-14-00351-s001.zip › Supplemental S6 Conditions in Calves.pdf]

Supplemental Table S6: Pain scoring for different conditions presented to participants of surveys about pain assessment in calves, under the assumption that no analgesics are used. Use of analgesics was not stated for [13,18,19,21]. Pain scoring was either done by veterinarians (V), veterinary students (VS), farmers (F), and/or producers (and frontline staff, P) by use of a Numerical Rating (NRS, ranging from either 0 – 10 or 1 – 10) or Visual Analogue Scale (VAS, ranging from 0 – 10), or both. [19] described using a NRS in the Material and Methods section, but indicated use of a VAS in the results section and is therefore included as VAS. Ranges are included in brackets if indicated in the references. If procedures were not presented in the respective reference, this is indicated as -.

	**NRS 0 - 10**						**NRS (1 – 10)**			**VAS (0 – 10)**		
Reference	[9]	[12]	[22][1]	[11]	[14]	[16][1]	[10][1]	[15]	[1]	[15]	[19]	[20]
Group	V	V	V/VS	V	F	V/F/P	V	VS	V	VS	F	P
Contracted tendons	-	-	-	4<br>(0 – 9)	4<br>(0 – 9)	-	-	-	-	-	-	-
(Broncho)Pneumonia	6<br>(1 – 10)	8<br>(2 – 10)	-	6<br>(1 – 10)	6<br>(1 – 10)	-	-	6<br>(2 – 10)	7<br>(1 – 10)	6<br>(1 – 10)	6.7<br>(2 – 10)	-
Distal limb fracture[2]	8<br>(2 – 10)	-	9 ± 1.2	8<br>(3 – 10)	8<br>(3 – 10)	-	-	8<br>(4 – 10)	9<br>(5 – 10)	8<br>(4 – 10)	7.6<br>(0 – 10)	-
Enteritis/Diarrhea[3]	-	-	-	5<br>(1 – 10)	5<br>(1 – 10)	4.6 ± 2.5	-	-	6<br>(1 – 10)	5<br>(1 – 10)	4.8<br>(1 – 10)	-
Following dystocia[4]	4<br>(1 – 10)	3<br>(1 – 10)	5.9 ± 1.9	5<br>(0 – 10)	5<br>(0 – 10)	-	-	4<br>(1 – 8)	5<br>(1 – 9)	4<br>(1 – 8)	3.3<br>(0 – 10)	-
Ileus	-	-	-	9<br>(1 – 10)	9<br>(1 – 10)	-	-	-	-	-	-	-
Joint ill[5]	7<br>(1 – 10)	8<br>(2 – 10)	-	8<br>(1 – 10)	8<br>(1 – 10)	-	-	7<br>(3 – 9)	7<br>(3 – 10)	6<br>(2 – 9)	6.7<br>(1 – 10)	-
Meningitis	-	-	-	8<br>(0 – 10)	8<br>(0 – 10)	-	-	-	-	-	-	-
Navel infection	-	-	6.8 ± 1.6	7<br>(2 – 9)	7<br>(2 – 9)	-	-	5<br>(2 – 8)	-	5<br>(1 – 9)	5.2<br>(1 – 10)	8
Needle prick neck	-	-	2.4 ± 1.6	-	-	-	-	-	-	-	-	-
Ruminal acidosis	-	-	-	4<br>(0 – 9)	4<br>(0 – 9)	-	-	-	-	-	-	-
Umbilical abscess	5<br>(1 – 10)	5<br>(1 – 9)	-	-	-	-	-	-	6<br>(2 – 9)	-	-	-
Umbilical hernia[6]	-	-	-	-	-	-	-	-	-	-	-	6

[1] mean values including standard deviation where indicated; [2] broken bone with open fracture on calf's hind leg for Norring et al. (2014), fracture of long bone for Tschoner et al. (2020, 2021); 3 intestinal infection for Kielland et al., 2009; 4 fetal-maternal disproportion requiring traction alone for Huxley and Whay (2006), Tschoner et al. (2020, 2021), strong pull assistance for Norring et al. (2014); 5 septic arthritis/polyarthritis for Tschoner et al. (2020, 2021); [6] the size of an apple for Wikman et al. (2013);
